# Attributes Underlying Non-surgical Treatment Choice for People With Low Back Pain: A Systematic Mixed Studies Review

**DOI:** 10.34172/ijhpm.2020.49

**Published:** 2020-04-08

**Authors:** Thomas G. Poder, Marion Beffarat

**Affiliations:** ^1^School of Public Health, University of Montreal, Montreal, QC, Canada.; ^2^Research Center of the IUSMM, CIUSSS de l’Est de l’Île de Montréal, Montreal, QC, Canada.; ^3^CERDI, Université Clermont Auvergne, ClermontFerrand, France.

**Keywords:** Low Back Pain, Preference, Treatment, Choice, Systematic Review

## Abstract

**Background:** The knowledge of patients’ preferences in the medical decision-making process is gaining in importance. In this article we aimed to provide an overview on the importance of attributes underlying the choice of non-surgical treatments in people with low back pain (LBP).

**Methods:** A systematic mixed studies review was conducted. Articles were retrieved from the search engines PubMed, ScienceDirect, and Scopus through June 21, 2018. The Mixed Methods Appraisal Tool (MMAT) was used to assess the quality of the study, and each step was performed by 2 reviewers.

**Analysis:** From a total of 390 articles, 13 were included in the systematic review, all of which were considered to be of good quality. Up to 40 attributes were found in studies using various methods. Effectiveness, ie, pain reduction, was the most important attribute considered by patients in their choice of treatment. This attribute was cited by 7 studies and was systematically ranked first or second in each. Other important attributes included the capacity to realize daily life activities, fit to patient’s life, and the credibility of the treatment, among others.

**Discussion:** Pain reduction was the most important attribute underlying patients’ choice for treatment. However, this was not the only trait, and future research is needed to determine the relative importance of the attributes.

## Introduction

Low back pain (LBP) is a common condition experienced by most individuals at least once during their lifetime.^1,2^ LBP refers to pain located between the lower rib margins and the buttock creases.^3^ Generally, the lower back is where most back pain occurs. According to the National Institute of Neurological Disorders and Strokes,^4^ a branch of the National Institute of Health, chronic LBP is defined “as pain that persists for 12 weeks or longer.”

In industrialized countries, the prevalence of LBP in a person’s lifetime was assessed at 60% to 70%^5^ and the incidence rate was between 60% and 90%.^6^ An evolution toward chronicity of LBP was observed in 6 to 8% of cases.^7,8^ Throughout the world, chronic LBP has high economic/professional (incapacity, absenteeism, activity limitation) and social (isolation, decrease in quality of life, constant need of care) impact on the population. Indeed, chronic LBP is the second cause of incapacity after cardiovascular disease.^9^ To effectively treat this population is essential. However, to be effective, these treatments must adhere to patients’ concerns, values and beliefs, and thus, consider their preferences.^10^

According to Bowling and Ebrahim,^11^ treatment preference is defined as the option chosen by the patient after having assessed the risks and benefits of available actions. To take into account the preference of patients in their choice of treatment is especially important in LBP, considering the large number of potential treatments, ie, more than 200 according to Haldeman and Dagenais,^12^ and their relatively low effectiveness.^13^ In addition, Aboagye^14^ puts forward other reasons for which preferences need to be examined in the treatment of this specific condition, including patient empowerment and satisfaction.

According to the Common Sense Model,^15^ a widely used theoretical framework to explain the processes by which patients become aware of and interact with a health threat, patients develop treatment preferences when attempting to match their illness representations with treatment beliefs. Therefore, it is important to consider what drives their choice for treatment and to better understand their preferences for the various attributes (ie, characteristics) describing a given treatment. This is also highlighted by Aboagye^14^ and the National Institute for Health and Care Excellence,^16^ who indicate that preferences and individual values are important and must be considered in the intervention choice process.

To contribute to a better understanding of which preferences drive treatment choice in LBP patients, we conducted a systematic mixed studies review. Specifically, the purpose of this article is twofold: (1) to determine which non-surgical treatment attributes are important for patients in their decision-making process, and (2) to report the ranking of these attributes in order of patients’ preferences.

## Methods

A systematic mixed studies review of the literature was conducted on non-surgical treatment preferences of people with LBP. To do so, we followed the statement rules used in our health technology assessment unit (unpublished), which are very close to what is described in the guideline developed for systematic reviews by the Institut national d’excellence en santé et en services sociaux (INESSS),^17^ the national health technology assessment agency in Quebec, Canada. The rational for a systematic mixed studies review was to get as much information as possible on this specific topic which may have been understudied. In addition, studying attributes that drive non-surgical treatment preferences will help decision-makers in our institution to reorganize the patients’ trajectory of care and to offer patients alternatives to surgical care. The methodological quality of each study was evaluated using the Mixed Methods Appraisal Tool (MMAT).^18^ In our review protocol, the inclusion criteria were established so as to be as exhaustive as possible. These criteria included studies analyzing health preferences regardless of the method used, eg, discrete choice experiment (DCE), qualitative studies, mix method design, ranking studies, swing weighting studies, analytical hierarchy process, and best-worst scaling. We also used studies referring to acute or chronic pain treatments in the low back region. Exclusion criteria were: preferences other than those of patients, sub-studies of other studies, studies about utilities associated with any health condition, studies combining data from patients with pain other than in the low back region, and studies that only referred to surgical treatment (ie, a study could compare surgical treatment with non-surgical treatment, but could not compare two surgical treatments). There was no limitation of language.

As per protocol, inclusion and exclusion criteria were established before conducting searches in the electronic database and were applied to the final search field. The search engines used in this systematic review were PubMed, ScienceDirect, and Scopus. In addition, to consider unpublished studies we completed the review by scanning references of included studies and contacted the authors who had performed a literature review prior to conducting their research. However, we did not perform a specific search in the grey literature. The search was conducted without date limits through June 21, 2018, using combinations of key search terms such as: “low back pain,” “lumbosacral region,” “health preference,” “patient preference,” “stated preference,” “stated choice,” and “treatment.” The complete search strategy based on keywords is available in Supplementary file 1.

Two reviewers (TGP and MB) independently screened the titles and abstracts (first phase of selection) using the criteria. If the criteria were met, the article was selected for a full reading (second phase of selection). The complete readings as well as the scoring with the MMAT were carried out by the 2 independent reviewers. After a full reading, articles were included if they corresponded to inclusion and exclusion criteria. At each step, disagreements were solved with an arbitration performed by a third reviewer. For both phases of selection, Cohen’s kappa coefficients were calculated to measure the degree of agreement. The value of the coefficients can be interpreted as follows: values ≤0 indicated no agreement; 0.01–0.20, none to slight; 0.21–0.40, fair; 0.41– 0.60, moderate; 0.61–0.80, substantial; and 0.81–1.00 was almost perfect agreement. Data were extracted by 1 reviewer (MB) and a second reviewer (TGP) checked and completed this data for accuracy. Any additional information added in the extraction grid was discussed between the 2 reviewers and disagreements were solved by the arbitration of a third reviewer. The main variables of interest in this systematic review were the preferences attributes and their levels. The following variables were also systematically collected: country, type of study, type of treatment, numbers of patients and their characteristics, results as a ranking or a size effect, type of statistical analysis, and other available characteristics, such as the recruitment process and the nature of the treatment experienced. Authors were contacted when data could not be retrieved from the selected articles. The data collected were examined and found to be inappropriate for a meta-analysis considering the high heterogeneity in the study designs and results (ie, different methods to assess preferences, different choice and definition of attributes and levels, different ways to report results). The relative importance of attributes was reported according to the ranking provided by the authors of the included studies.

## Results

In total, 390 studies were identified after the removal of duplicates, 37 of which were fully read to assess their eligibility. A total of 13 studies were selected to be included in the systematic mixed studies review. The Cohen’s kappa coefficient was 0.7937 in the first phase of the selection process (screening of both titles and abstracts) and 0.9217 in the second phase (full-text readings). The reasons for excluding 24 studies that were fully read were as follows: the study was a systematic review without original data (n = 3)^19-21^; the study did not consider the preferences of patients (n = 4)^13,14,22,23^; the study analyzed preferences but not for treatment characteristics (n = 11)^24-34^; the pain site was somewhere other than in the low back or data were aggregated with other sites (n = 4)^35-38^; the study was a sub-study of another one (n = 1)^39^; and data was not available even after contacting the authors (n = 1)^40^. Details of the process selection can be found in the Preferred Reporting Items for Systematic Reviews and Meta-Analyses (PRISMA) flow diagram in Figure.

**Figure:**
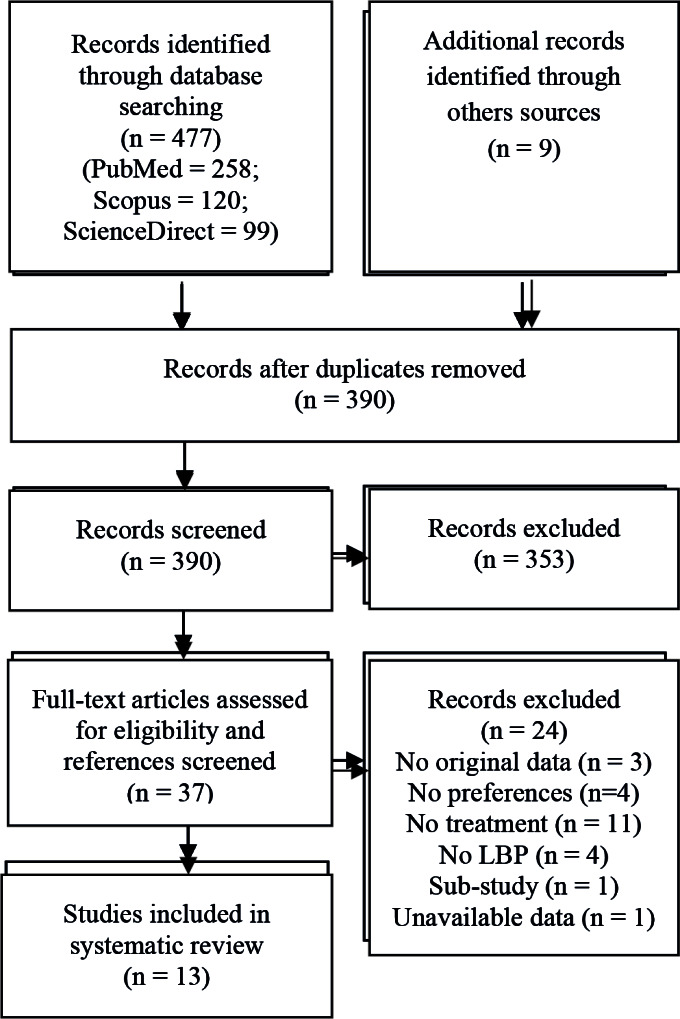
PRISMA Flow Diagram. Abbreviations: PRISMA, Preferred Reporting Items for Systematic Reviews and Meta-Analyses; LBP, low back pain.

Table 1 lists the 13 selected studies.^41-53^ A majority of these studies (n = 7) were published during the past 5 years and mainly originated from Europe (n = 7) and the United States (n = 3). This shows that the topic of health preferences is increasingly gaining importance in the Western world. Very few information about the characteristics of the respondents were provided in the studies, with the exception of age and gender. Of the 11 studies that reported these data, mean age ranged from 41 to 62 years, and mean proportion of women was between 50.4% and 75.6%. Seven of the included studies were qualitative, while the others were mixed-method or quantitative studies, including 4 DCEs. In general, included studies had a satisfactory score of quality. None of these studies had a score below 50% in the MMAT. In addition, studies with lower scores were mainly because of missing information in their method’s section. As a result, the MMAT score had little impact on how to interpret the findings. A very high heterogeneity in study designs was observed in this systematic review. In particular, the primary studies each used specific measurement methods for patients’ preferences. Some were measured with questionnaires and others used focus groups or individual interviews, while the DCE studies used different attributes and levels for treatments. This precluded performing a meta-analysis.

**Table 1:** Characteristics of Studies Included in the Systematic Review

**Authors/Year**	**Country**	**Study’s Method**	**No. of Patients**	**MMAT Score**	**Source of Funding**
François et al/2018	USA	Quantitative (cross-section)	104	68.75%	NIHR, NICHD, NCMRR
Aboagye et al/2017	Sweden	Quantitative (DCE)	112	95.85%	AFA Insurance, Swedish Research Council for Health, Working Life and Welfare
Verbrugghe et al/2017	Belgium	Mixed method (interviews questionnaires)	40	58%	Not declared
Chen et al/2015	China	Quantitative (DCE)	86	75%	Research Committee of the University of Macau
Dima et al/2015	England	Quantitative (questionnaires)	115	70.5%	NIHR School for Primary Care Research
Gardner et al/2015	Australia	Qualitative (Interviews)	20	70.83%	Self-financing
Klojgaard et al/2014	Denmark	Quantitative (DCE)	348	83.35%	Danish Strategic Research Council project CeSpine
Dima et al/2013	England	Qualitative (focus group)	75	81.25%	NIHR School for Primary Care Research
Haanstra et al/2013	USA	Qualitative (interviews)	77	77.1%	Not declared
Klojgaard et al/2012	Denmark	Qualitative (interviews)	3	91.65%	Danish Strategic Research Council project CeSpine
Yi et al/2011	Scotland	Quantitative study (DCE)	124	62.5%	Scottish Government Health Directorate and Aberdeen University
Hsu et al/2010	USA	Qualitative (interviews)	327	64.62%	NIH-NCCAM, NIAMSD
Slade et al/2009	Australia	Qualitative (focus group)	18	58.35%	National Health and Medical Research Council PhD Scholarship

Abbreviations: MMAT, Mixed Methods Appraisal Tool – the score provided is the mean of both reviewers; DCE, discrete choice experiment; NIHR, National Institute for Health Research; NICHD, National Institute of Child Health and Human Development; NCMRR, National Center for Medical Rehabilitation Research; NIH, National Institute for Health; NCCAM, National Center for Complementary and Alternative Medicine; NIAMSD, National Institute for Arthritis and Musculoskeletal and Skin Disease.

Results of the systematic mixed studies review are reported in Table 2. According to studies included in this review, the attributes most frequently cited in the preferences of patients were effectiveness (ie, reduction in pain level), the capacity to realize daily life activities, fit to the patient’s life, providers’ attitudes and characteristics, and the frame/design of the treatment (eg, supervised or not, in groups or individually). These attributes were cited in at least four studies. Among these five attributes, effectiveness and capacity to realize daily life activities appeared to be the most valued, while providers’ attitudes and characteristics seemed to be much less important.

**Table 2:** Preferences of Patients for Each Attribute of Treatments

**Attribute**	**Importance/Ranking**	**Treatment Modality (Levels)**	**Reference/Year**
Effectiveness/pain reduction	Relevant (determined during focus group) Same weight but prioritised by patients, top 4 Relevant (determined during focus group) Same weight but prioritised by authors, top 4 Relevant (validated questionnaire) Same weight – ranked 2-4 over 4 attributes Significant *P* < .001 – ranked 2/4 Significant *P* < .001 – ranked 1/4 Relevant (determined by literature review, doctors and patients) – ranked 1-5/17 Relevant (determined by patients’ interviews) – ranked 1/9	Six different treatments Dx, exercise, manual therapy, acupuncture Exercise Acupuncture, infrared treatment (minor, moderate, major reduction) Surgical vs. non-surgical (same, less, none) Surgical vs. non-surgical HDS or home exercise, spinal manipulation	Dima et al/2013 Dima et al/2015 François et al/2018 Chen et al/2015 Klojgaard et al/2014 Klojgaard et al/2012 Haanstra et al/2013
Capacity to realize common/leisure/daylife activities	Relevant (determined by patients) – ranked in top 3 Significant *P* < .001 (positive) – ranked 2/4 Relevant (determined by patients’ interviews) – ranked 2/9 Relevant (determined by literature review, doctors and patients) – ranked 1-5/17	Rehabilitation program + exercise Surgical vs. non-surgical (same, fewer, none) HDS or home exercise, spinal manipulation surgical vs. non-surgical	Verbrugghe et al/2016 Klojgaard et al/2014 Haanstra et al/2013 Klojgaard et al/2012
Fit to patients’ life/convenience	Relevant (determined during focus group) same weight, top 4 Relevant (determined during focus group) same weight, top 4 Relevant (validated questionnaire) most important according to authors – ranked 1/4 Relevant (determined during focus group) time management and flexible time-tables for 18/18 persons, fit to patients’ capacities for 18/18 persons	Six different treatments Dx, exercise, manual therapy, acupuncture Exercise Physical exercises program	Dima et al/2013 Dima et al/2015 François et al/2018 Slade et al/2009
Frame/design of the treatment (supervision or not and individual or group)	Significant *P* < .001 for group with supervision – attribute ranked 4/6 – weight 17% Relevant (determined during focus group) Non-clinical setting for 16/18 persons, close supervision for 16/18 persons and in group for 11/18 persons Significant *P* < .01 preference for small group – ranked 1/5 Relevant (determined by patients’ interviews) 9/9	Exercise (Individual w/o supervision, group w/o supervision) Physical exercises program Pain management program (individual, 2-6, 7-12, more than 12) HDS or home exercise, spinal manipulation	Aboagye et al/2017 Slade et al/2009 Yi et al/2011 Haanstra et al/2013
Providers’ attitudes and characteristics	Relevant (determined by patients’ interviews) – ranked 9/9 Relevant (determined during focus group) encouraging instructors and their quality teaching skills, take time to listen and shared decision-making for 18/18 persons Relevant (determined by focus group) conscientious, knowledgeable, empathic, respectful and trustworthy, outside the top 4 Non-significant – ranked 3/5	HDS or home exercise, spinal manipulation physical exercises program Six different treatments Pain management program (nurse, pharmacist, physiotherapist, GP, psychologist, pain team)	Haanstra et al/2013 Slade et al/2009 Dima et al/2013 Yi et al/2011
Credibility of treatment	Relevant (determined during focus group) Same weight, top 4 Relevant (determined during focus group) Same weight but prioritised by authors, top 4 Relevant (determined by patients’ interviews) Awareness and Confidence in treatment options – ranked 1/11 – weight 16.2%	Six different treatments Dx, exercise, manual therapy, acupuncture CAM	Dima et al/2013 Dima et al/2015 Hsu et al/2010
Capacity to return to work	Relevant (determined by patients) – ranked 2/5 – weight 14.29% Relevant (determined by patients) ranked in top 3 Relevant (determined by literature review, doctors and patients) – ranked 6-17/17	Physiotherapy Rehabilitation program + exercise Surgical vs. non-surgical	Gardner et al/2015 Verbrugghe et al/2016 Klojgaard et al/2012
Treatment frequency	Significant *P* < .001 for Once or two times per week – attribute ranked 3/6 – weight 18% Significant *P* < .01 preference for fewer sessions over a longer period – ranked 2/5 Relevant (determined by literature review, doctors and patients) – ranked 6-17/17	Exercise (once, 2, 3 per week) Pain management program (10, 5, 2, 1 sessions a week over 2, 4, 10, 20 weeks) Surgical vs. non-surgical	Aboagye et al/2017 Yi et al/2011 Klojgaard et al/2012
Onset of treatment efficacy	Significant *P* < .001 – ranked 4/4 Significant *P* < .001(negative) – not ranked, used as reference Relevant (determined by literature review, doctors and patients) – ranked 1-5/17	Acupuncture, infrared treatment (2, 4, 8 courses) Surgical vs. non-surgical (1, 3, 6, 12 months) Surgical vs. non-surgical	Chen et al/2015 Klojgaard et al/2014 Klojgaard et al/2012
Content of program/treatment	Non-significant except for education + drug management *P* < .05 (negative) – ranked 5/5 Relevant (determined by patients’ interviews) – ranked 7/9 Relevant (determined by literature review, doctors and patients) – ranked 6-17/17	Pain management program (education, physical therapy, coping with pain, drug management) HDS or home exercise, Spinal manipulation surgical vs. non-surgical	Yi et al/2011 Haanstra et al/2013 Klojgaard et al/2012
Energy/ability to sleep	Relevant (determined by patients) – ranked 5/5 – weight 6.35% Relevant (determined by patients’ interviews) – ranked 8/11 – weight 2.4% Relevant (determined by literature review, doctors and patients) 6-17/17	Physiotherapy CAM Surgical vs. non-surgical	Gardner et al/2015 Hsu et al/2010 Klojgaard et al/2012
Realize physical activities	Relevant (determined by patients) – ranked 1/5 – weight 49.2% Relevant (determined by literature review, doctors and patients) – ranked 6-17/17	Physiotherapy Surgical vs. non-surgical	Gardner et al/2015 Klojgaard et al/2012
Type of exercise	Significant *P* < .001 for cardiovascular training – attribute ranked 2/6 – weight 19% Relevant (determined during focus group) Fun and varied exercises for 18/18 persons, water-based for 8/18	Exercise (cardiovascular, strength, mindfulness-based training) Physical exercises program	Aboagye et al/2017 Slade et al/2009
Risk of relapse	Significant *P* < .001 for 30% risk (negative) – ranked 3/4 Relevant (determined by literature review, doctors and patients) – ranked 1-5/17	Surgical vs. non-surgical (10%, 20%, 30%) Surgical vs. non-surgical	Klojgaard et al/2014 Klojgaard et al/2012
Patients’ concerns (financial and security)	Relevant (determined during focus group) same weight, top 4 Relevant (determined during focus group) same weight, top 4	Six different treatments Dx, exercise, manual therapy, acupuncture	Dima et al/2013 Dima et al/2015
Improvement in emotional state	Relevant (determined by patients’ interviews) Emotional state ranked 3/11 – weight 8.3% - Well-being ranked 6/11 – weight 3.5% Relevant (determined by literature review, doctors and patients) – ranked 6-17/17	CAM Surgical vs. non-surgical	Hsu et al/2010 Klojgaard et al/2012
To have a social life	Relevant (determined by patients) – ranked 4/5 – weight 6.35% Relevant (determined by literature review, doctors and patients) – ranked 6-17/17	Physiotherapy Surgical vs. non-surgical	Gardner et al/2015 Klojgaard et al/2012
Out-of pocket cost	Significant *P* < .001 – not ranked, used as reference Relevant (determined by focus group) for 10/18 persons	Acupuncture, Infrared treatment (120, 600, 1000 CNY per course) Physical exercises program	Chen et al/2015 Slade et al/2009
Knowledge about their body	Relevant (determined by patients’ interviews) ranked 4/11 – weight 7.6% Relevant (determined by focus group) for 18/18 persons	CAM Physical exercises program	Hsu et al/2010 Slade et al/2009
Knowledge about treatment and disease	Relevant (determined by patients’ interviews) – ranked 5/9 Relevant (determined by focus group) for 18/18 persons	HDS or home exercise, spinal manipulation physical exercises program	Haanstra et al/2013 Slade et al/2009
Knowledge about etiology and access to real diagnostic	Relevant (determined by patients’ interviews) – ranked 6/9 Relevant (determined during focus group), outside the top 4	HDS or home exercise, spinal manipulation six different treatments	Haanstra et al/2013 Dima et al/2013
Self-management capacities	Relevant (determined by patients’ interviews) – ranked 3/9 Relevant (determined by focus group), outside the top 4	HDS or home exercise, spinal manipulation six different treatments	Haanstra et al/2013 Dima et al/2013
Others symptoms non related to LBP	Relevant (determined by researchers, doctors and patients) – ranked 6-17/17 Relevant (determined by patients’ interviews) – ranked 7/11 – weight 2.7%	Surgical vs. non-surgical CAM	Klojgaard et al/2012 Hsu et al/2010
Proximity	Non-significant – attribute ranked 6/6 – weight 4% Significant *P* < .01 (negative) – ranked 4/5	Exercise (10, 20, 30 minutes) Pain management program (15, 30, 45, 60, 75, 90, 105, 120 minutes from the clinic)	Aboagye et al/2017 Yi et al/2011
Incentives	Significant *P* < .001 for none, exercise at work and wellness subsidies – attribute ranked 5/6 – weight 17%	Exercise (none, wellness subsidies, exercise at work, discount coupon)	Aboagye et al/2017
Exercise intensity	Significant *P* < .001 for High intensity – attribute ranked 1/6 – weight 25%	Exercise (low, high, medium)	Aboagye et al/2017
Acceptability/logicality	Relevant (validated questionnaire) same weight – ranked 2-4 over 4 attributes	Exercise	François et al/2018
Suitability/appropriateness	Relevant (validated questionnaire) same weight – ranked 2-4 over 4 attributes	Exercise	François et al/2018
Knowledge of the exercise	Relevant (determined during focus group) for 18/18 persons	Physical exercises program	Slade et al/2009
Duration of efficacy	Significant *P* < .001 – ranked 3/4	Acupuncture, Infrared treatment (2, 6, 12 months)	Chen et al/2015
Sensation of treatment	Significant *P* < .001 – ranked 1/4	Acupuncture, Infrared treatment (sore and numb, mild thermal and vibration)	Chen et al/2015
Find motivation and self-confidence	Relevant (determined by patients’ interviews) 8/9	HDS or Home exercise, Spinal manipulation	Haanstra et al/2013
Improvement biomechanical functioning	Relevant (determined by patients’ interviews) – ranked 4/9	HDS or Home exercise, Spinal manipulation	Haanstra et al/2013
Relaxation (mind and body)	Relevant (determined by patients’ interviews) relaxation ranked 2/11 – weight 8.3% - mind-body-spirit ranked 10/11 – weight 1.1% - mindfulness ranked 11/11 – weight 0.5%	CAM	Hsu et al/2010
Changes in way of thinking	Relevant (determined by patients’ interviews) ranked 5/11 – weight 4.9%	CAM	Hsu et al/2010
Dramatic improvement in overall health and well-being	Relevant (determined by patients’ interviews) ranked 9/11 – weight 1.5%	CAM	Hsu et al/2010
Use of pain killers	Relevant (determined by literature review, doctors and patients) – ranked 6-17/17	Surgical vs. non-surgical	Klojgaard et al/2012
Neurological deficits	Relevant (determined by literature review, doctors and patients) – ranked 6-17/17	Surgical vs. non-surgical	Klojgaard et al/2012
Coping skills	Relevant (determined by patients) – ranked 3/5 – weight 11.11%	Physiotherapy	Gardner et al/2015
Seeking alternative treatment	Relevant (determined by focus group), outside the top 4	Six different treatments	Dima et al/2013

Abbreviations: w/o, with or without; Dx, medication; CAM, complementary and alternative medicine; HDS, high dose supervised; GP, general practitioner; LBP, low back pain. Difference between relevant and significant is related to the use of a statistical test or not.

Alternatively, other attributes were less frequently cited but revealed strong preferences. This was particularly the case for credibility of treatment, capacity to return to work, and treatment frequency. These three attributes were cited in three studies each. Other attributes were also cited in three studies, but revealed less important preferences: onset of treatment efficacy, content of program, and energy/ability to sleep. Other attributes were only considered in one or two studies, thus making it difficult to identify which elements were really important for patients when choosing a treatment (see Table 2).

Some attributes provided conflicting results. This was particularly the case for the frame/design of the treatment and for the onset of treatment efficacy. While close supervision appeared to be valued by patients, the optimal size of the group supervised is still to be determined. In regard to the onset of treatment efficacy, patients seemed willing to wait a long time if the treatment would meet their expectations (ie, effectiveness).

Patients’ preferences in term of treatment modality are reported in Table 3. One study did not compare treatments,^49^ considering only one treatment. Six studies only concerned the patients’ preferences of attributes and not their treatment preferences.^44-46,51-53^ Consequently, only six studies investigated a specific preference for one of the treatments.^41-43,47-48,50^ Surgical treatment and acupuncture seemed to be less frequently preferred than other alternatives, such as physical exercise and medication. Most studies were about physical activities and compared various types of exercise, but no obvious tendency appeared.

**Table 3:** Preferences in Terms of Treatment Modality

**Author, Year**	**Treatment Modality**	**Preference**
Francois et al, 2018	SF training, MST	SF training > MST Preferences before the intervention: 91.3% preferred SF and 8.7% MST. After the intervention: scores at 3.88 for SF and 3.58 for MST
Aboagye et al, 2017	Cardiovascular training, strength training, mindfulness-based training	Cardiovascular training > mindfulness-based training >strength training Significant at *P* < .001
Verbrugghe et al, 2017	Rehabilitation program (aerobe exercise therapy, posture correction, breathing control, stabilization exercises and home exercises)	No precise preference. Household related activities were the most preferred training activity
Chen et al, 2015	Infrared therapy, acupuncture	Infrared therapy >Acupuncture 47.5% choose infrared therapy against 43.9% who choose acupuncture
Dima et al, 2015	Medication, exercise, manual therapy, acupuncture	Exercise ≈ Medication >Manual therapy >Acupuncture Exercise 3.64 ≈ 3.63 medications, manual therapy 3.54, acupuncture 3.25. In a ranking exercise, 152 persons ranked medication first, whereas it was 88 for exercise, 89 for manual therapy and 24 for acupuncture
Gardner et al, 2015	Physiotherapy	No comparison with another treatment
Kløjgaard et al, 2014	Non-surgical and surgical interventions	Non-surgical > Surgical interventions Surgical interventions significant at *P* < .001 with negative preference
Dima et al, 2013	Medication, exercise, manual therapy, acupuncture, combined and psychological approach, spinal fusion	No preference assessed
Haanstra et al, 2013	High Dose Supervised Exercise, Home Exercise, Chiropractic spinal manipulation	No preference assessed
Kløjgaard et al, 2012	Non-surgical and surgical interventions	No preference assessed
Yi et al, 2011	Pain management program (education, physical therapy, coping with pain, medicines management)	No precise preference. Patients seemed to be against Education and Medicines Management when combined, significant at *P* < .01 with negative preference
Hsu et al, 2010	CAMs	No preference assessed
Slade et al, 2009	Physical exercises program	No precise preference. Some patients spontaneously cited water-based exercise (8/18)

Abbreviations: SF, strength and flexibility; MST, motor skill training; CAMs, complementary and alternative medicines. Note: When treatment A is preferred to treatment B, we indicated A > B.

## Discussion

We identified which non-surgical treatments attributes for LBP were preferred by patients based on the scientific literature. As previously indicated, treatment preference is the option a patient chooses after considering the risks and benefits of the multiple options available for treatment of a clinical condition.^11^ In this setting, treatment preference was led by the preferences of patients according to the attributes and expected benefits, which are on their turn based on their experiences, knowledge and beliefs about the treatment. Previous authors have suggested that including patients’ preferences in clinical decision-making about optimal treatment is a central aspect of practising evidence-based medicine.^11,54-55^ As such, to include patient preferences in the decision-making process has gained in importance among doctors.^14^ Knowing the patient’s general expectations and preferences not only guides the choice of treatment, but may potentially improve the outcome of the treatment.^56^ Moreover, patients want to be included in this process, which leads to greater satisfaction.^57,58^

According to this systematic review, the most frequently mentioned attributes in the preferences of patients for non-surgical treatments were effectiveness, capacity to realize daily life activities, fit to the patient’s life, providers’ attitudes and characteristics, and the frame/design of the treatment (eg, supervised or not, in groups or individually). However, being mentioned does not guarantee that these attributes are considered important for patients. Indeed, these attributes are not of equal importance. By far, effectiveness is the attribute most mentioned (ie, 7 studies of 13) and the one that is frequently given the highest consideration by LBP patients. Other important attributes were capacities to realize daily life activities, fit to the patient’s life, credibility of the treatment, capacity to return to work, and treatment frequency (ie, generally fewer sessions over a longer time period).

As per protocol, studies outside the scope of LBP were excluded from this systematic review. However, the results found are congruent with other chronic pain conditions.^35,37^ To our knowledge, this study is the first systematic review on the topic of LBP patients’ preferences for attributes of treatments underlying their choices. This study will be useful for future research in this field and especially for preparing new studies that aim to elicit the preferences of patients to offer them convenient healthcare services and to better fit the design of intervention toward LBP patients. Indeed, knowing the patient’s preference for a given treatment is not sufficient to improve healthcare quality. This is why we need to know which attributes are important in the choice of a treatment modality by patients. This will help in clinical practice on how to adapt the design of treatments to better fit patients’ preferences and incite patients to be more adherent. As an example, many studies have revealed that patients have preferences for home exercises, but have found that between 50% and 70% of chronic LBP patients did not perform these prescribed home exercises.^19^ As such, patients’ preferences for specific attributes of home exercise could potentially impact clinical outcomes through adherence.

Several limits rise from this systematic mixed studies review. First, all included studies did not determine patients’ preferences using the same method: a number were identified with focus groups, some with interviews and/or questionnaires, and others with DCEs using different attributes and levels. In addition, some studies used statistical tests to compare the attributes, while others studies simply considered the attributes given spontaneously by patients or asked patients to perform a ranking. This could be interpreted as a methodological limitation for this review and could impede the comparability between results. Second, not all studies used the same attributes, which makes the comparison of attributes between studies even harder. Third, we indirectly assessed the risk of bias of the included studies using the MMAT which is imperfect considering that this tool mostly evaluates the quality of mixed-methods studies. However, we are not aware of specific tools to assess the risk of bias in preference studies. Fourth, all reviews, including the present one, is limited by the search strategy and the selection of databases, which may have led to some missed studies. Fifth, preferences may vary across populations with disparate demographic characteristics, but due to limited data provided in the studies we were not able to assess if these characteristics have an impact on patients’ preferences. Sixth, some information is missing or insufficiently described in the studies retrieved, such as at what time in the consultation process the patients were asked for their preferences, the information they may have received about treatments, and data to determine if patients were comparable from one study to another. This information would have been helpful to better understand patients’ preferences. Seventh, we attempted to report the attributes by the main treatment modalities (eg, exercise, acupuncture, surgical vs. non-surgical), but no specific pattern was found. A potential explanation for this is that each modality, even in the same category, can differ greatly from each other. Finally, included studies had various objectives, which may have led to different rankings or even omitting certain attributes. Despite the fact that we conducted a rigorous selection process in this systematic review, all these points are strong limitations that preclude establishing a clear ranking as to patients’ preferences.

However, as said above, a strength of this review is that we followed a standard and rigorous method, thus allowing to find some key preferences in treatment attributes. Moreover, this review is in line with various international recommendations to consider patients’ views in order to improve patient-centered care.^59^ Although including patients in clinical decisions may be challenging, patient involvement may potentially have a significant effect on treatment outcomes.^60^ The benefits of patient involvement and the skills required to achieve this is thus a central aspect of practicing evidence-based medicine.^60^ In this sense, the present study is important as it aims to highlight patients’ treatment preferences, which is pertinent for caregivers to know.

## Conclusion

In this systematic mixed studies review, we found that effectiveness (ie, pain reduction) was the most important attribute considered by patients in their choice of a treatment. This attribute was cited in seven of the thirteen included studies and was systematically ranked first or second. Other important attributes were the capacity to realize daily life activities, fit to the patient’s life, and credibility of the treatment, among others. However, these are not the only traits and future research is needed to clearly determine their relative importance. This research is important considering that patients’ preference is essential in the decision-making process, since it could influence adherence to treatment and clinical outcomes. This is part of a process whereby healthcare providers should share treatment decisions with patients by listening to them, trying to understand them, and considering their wishes.^50^

## Acknowledgement

We acknowledge the UETMISSS team at the CIUSSS de l’Estrie – CHUS. TGP is member of the FRQS-funded Centre de recherche de l’IUSMM.

## Ethical issues

This article does not contain any studies with human participants performed by any of the authors.

## Competing interests

Authors declare that they have no competing interests.

## Authors’ contributions

TGP and MB conceived and conducted the study. TGP wrote the manuscript and MB revised it critically.

## Authors’ affiliations

^1^School of Public Health, University of Montreal, Montreal, QC, Canada. ^2^Research Center of the IUSMM, CIUSSS de l’Est de l’Île de Montréal, Montreal, QC, Canada. ^3^CERDI, Université Clermont Auvergne, Clermont-Ferrand, France.

## Supplementary files

Supplementary file 1 contains the complete search strategy based on keywords.
