# Supplementary material for: Attributes Underlying Non-surgical Treatment Choice for People With Low Back Pain: A Systematic Mixed Studies Review
Source: Int J Health Policy Manag. 2020 Apr 8;10(4):201–10. doi: 10.34172/ijhpm.2020.49 (PMC8167275; doi:10.34172/ijhpm.2020.49)
Supplement: Supplementary file 1 — contains the complete search strategy based on keywords. [file ijhpm-10-201-s001.pdf]

Supplementary file 1

Search strategies on June 21, 2018.

PubMed

(((((((((("Patient Preference"[Mesh]) OR ((((Preference[Title/Abstract]) OR Preferences[Title/Abstract]) OR Prefer[Title/Abstract]) OR Preferring[Title/Abstract]))) OR (("Choice Behavior"[Mesh]) OR ((((Choice Behavior[Title/Abstract]) OR Choice Behaviors[Title/Abstract]) OR Approach Behavior[Title/Abstract]) OR Approach Behaviors[Title/Abstract])))) OR ((((((choice[Title]) OR choices[Title]) OR choosing[Title]) OR choose[Title]) OR chosen[Title]) OR chose[Title]))) AND ((((("Low Back Pain"[Mesh]) OR "Low Back Pain"[Title/Abstract]) OR "Low Back Pains"[Title/Abstract])) OR ((((((("Chronic Pain"[Mesh]) OR Chronic Pain[Title/Abstract]) OR Chronic Pains[Title/Abstract]) OR Chronic aches[Title/Abstract]) OR CLBP[Title/Abstract])) AND (((((("Lumbosacral Region"[Mesh]) OR lumbosacral[Title/Abstract]) OR lumbar[Title/Abstract]) OR sacral[Title/Abstract]) OR low back[Title/Abstract]) OR lower back[Title/Abstract])))) AND ((((((((((((("Therapeutics"[Mesh]) OR Therapeutic[Title/Abstract]) OR Therapeutics[Title/Abstract]) OR "Drug Therapy"[Mesh]) OR Therapy[Title/Abstract]) OR Therapies[Title/Abstract]) OR Treatment[Title/Abstract]) OR Treatments[Title/Abstract]) OR Pharmacotherapy[Title/Abstract]) OR Pharmacotherapies[Title/Abstract]) OR Pharmacologic[Title/Abstract])) OR (((((((("Conservative Treatment"[Mesh]) OR Conservative[Title/Abstract]) OR "non-invasive"[Title/Abstract]) OR noninvasive[Title/Abstract]) OR Nonoperative[Title/Abstract]) OR "Non-operative"[Title/Abstract]) OR nonsurgical[Title/Abstract]) OR "non-surgical"[Title/Abstract])) OR (((((((((("Pain Management"[Mesh]) OR Management[Title/Abstract]) OR Managements[Title/Abstract]) OR "Treatment Outcome"[Mesh]) OR Outcome[Title/Abstract]) OR Outcomes[Title/Abstract]) OR Clinical Effectiveness[Title/Abstract]) OR Clinical Effectivenesses[Title/Abstract]) OR "Clinical Efficacy"[Title/Abstract]) OR "Clinical Efficacies"[Title/Abstract]))))

SCOPUS

(((((TITLE-ABS-KEY (chronic PRE/3 pain) OR TITLE-ABS-KEY (chronic PRE/3 aches) OR TITLE-ABS-KEY (clbp) OR TITLE-ABS-KEY ({Low Back Pain}) OR TITLE-ABS-KEY ({Low Back Pains}))) AND ((TITLE-ABS-KEY ({Lumbosacral Region}) OR TITLE-ABS-KEY (lumbosacral) OR TITLE-ABS-KEY (lumbar) OR TITLE-ABS-KEY (sacral) OR TITLE-ABS-KEY (low PRE/2 back) OR TITLE-ABS-KEY (lower PRE/2 back)))) AND ((TITLE-ABS-KEY (therapeutic) OR TITLE-ABS-KEY (drug W/3 therapy) OR TITLE-ABS-KEY (therapy) OR TITLE-ABS-KEY (therapies) OR TITLE-ABS-KEY (treatment) OR TITLE-ABS-KEY (pharmacotherapy) OR TITLE-ABS-KEY (pharmacotherapies) OR TITLE-ABS-KEY (pharmacologic) OR TITLE-ABS-KEY (conservative) OR TITLE-ABS-KEY ({non-invasive}) OR TITLE-ABS-KEY ({non invasive}) OR TITLE-ABS-KEY (noninvasive) OR TITLE-ABS-KEY (nonoperative) OR TITLE-ABS-KEY ({Non-operative}) OR TITLE-ABS-KEY ({Non operative}) OR TITLE-ABS-KEY (nonsurgical) OR TITLE-ABS-KEY ({non-

surgical}) OR TITLE-ABS-KEY ({non surgical}) OR TITLE-ABS-KEY (management) OR TITLE-ABS-KEY (outcome) OR TITLE-ABS-KEY (clinical PRE/3 effectiveness) OR TITLE-ABS-KEY (clinical PRE/3 effectivenesses) OR TITLE-ABS-KEY ({Clinical Efficacy}) OR TITLE-ABS-KEY ({Clinical Efficacies})))) AND (((TITLE-ABS-KEY (preference) OR TITLE-ABS-KEY (prefer) OR TITLE-ABS-KEY (preferring))) OR ((TITLE-ABS-KEY (choice PRE/3 behavior) OR TITLE-ABS-KEY (approach PRE/3 behavior) OR TITLE (choice) OR TITLE (choosing) OR TITLE (choose) OR TITLE (chosen) OR TITLE (chose))))) AND NOT (INDEX (medline) OR PMID (1*) OR PMID (2*) OR PMID (3*) OR PMID (4*) OR PMID (5*) OR PMID (6*) OR PMID (7*) OR PMID (8*) OR PMID (9*))

ScienceDirect

(TITLE-ABSTR-KEY (Preference OR Preferences OR Prefer OR Preferring OR "Choice Behavior" OR "Choice Behaviors" OR "Approach Behavior" OR "Approach Behaviors") OR TITLE (choice OR choices OR choosing OR choose OR chosen OR chose)) AND (TITLE-ABSTR-KEY ("Low Back Pain" OR "Low Back Pains" OR "Chronic Pain" OR "Chronic Pains" OR "Chronic aches" OR CLBP) AND TITLE-ABSTR-KEY (lumbosacral OR lumbar OR sacral OR "low back" OR "lower back")) AND TITLE-ABSTR-KEY (Therapeutic OR Therapeutics OR Therapy OR Therapies OR Treatment OR Treatments OR Pharmacotherapy OR Pharmacotherapies OR Pharmacologic OR Conservative OR "non-invasive" OR noninvasive OR Nonoperative OR "Non-operative" OR nonsurgical OR "non-surgical" OR Management OR Managements OR Outcome OR Outcomes OR "Clinical Effectiveness" OR "Clinical Effectivenesses" OR "Clinical Efficacy" OR "Clinical Efficacies")
